# Efficacy and safety of aflibercept in in vitro and in vivo models of retinoblastoma

**DOI:** 10.1186/s13046-016-0451-7

**Published:** 2016-11-04

**Authors:** Dong Yoon Kim, Jeong A Choi, Jae-Young Koh, Young Hee Yoon

**Affiliations:** 1 Department of Ophthalmology, Chungbuk National University College of Medicine, Cheongju, Korea; 2 Neural Injury Research Center, Asan Institute for Life Sciences, University of Ulsan College of Medicine, Seoul, Korea; 3 Department of Neurology, Asan Medical Center, University of Ulsan College of Medicine, Seoul, Korea; 4 Department of Ophthalmology, Asan Medical Center, University of Ulsan College of Medicine, 88, Olympic-ro 43-Gil, Songpa-gu, Seoul, Korea

**Keywords:** Aflibercept, Retinoblastoma, Choroidal invasion, Treatment

## Abstract

**Background:**

To evaluate the inhibitory effects of aflibercept on the growth and subretinal invasion of retinoblastoma.

**Methods:**

Xenotransplantation and orthotopic mouse models were created by injecting Y-79 cells subcutaneously and intravitreally, respectively. After induction of retinoblastoma, animals were intraperitoneally injected with aflibercept (25 mg/kg body weight) or saline twice a week for 3 weeks. Tumor size was measured weekly and compared between the two groups. At 4 weeks, animals were sacrificed and an immunohistochemical examination was conducted to compare the microvascular density and degree of apoptosis between groups. In addition, the degree of choroidal invasion was also analyzed in the orthotopic xenotransplantation model. A co-culture system of Y-79 or WERI-Rb-1 cells and human umbilical vein endothelial cells (HUVECs) was used for in vitro experiments, and the anti-angiogenic effect of aflibercept was evaluated by analyzing cell numbers.

**Results:**

In the Y-79 xenotransplantation model, aflibercept treatment significantly inhibited tumor growth at 4 weeks versus baseline compared with saline-injected mice (188.53 ± 118.53 mm^3^ vs. 747.87 ± 118.83 mm^3^, respectively, *P* < 0.001). Tumors isolated from aflibercept-treated mice contained fewer blood vessels (8.59 % ± 7.60 % vs. 14.91 % ± 4.53 %, respectively, *P* < 0.05) and an increased number of apoptotic cells (15.10 ± 9.13 vs. 4.44 ± 2.24, respectively, *P* < 0.05). In the orthotopic model, the degree of subretinal invasion of tumor cells was significantly reduced after aflibercept treatment (0.07 ± 0.06 vs. 0.15 ± 0.10, *P* < 0.05). And addition of aflibercept to co-cultures of HUVECs and Y-79, WERI-Rb-1 cells significantly reduced HUVEC proliferation.

**Conclusions:**

Aflibercept reduced retinoblastoma angiogenesis in association with a significant reduction in tumor growth and invasion. These findings suggest that aflibercept could be used in an adjuvant role together with systemic chemotherapy to reduce tumor size and angiogenesis in retinoblastoma.

**Electronic supplementary material:**

The online version of this article (doi:10.1186/s13046-016-0451-7) contains supplementary material, which is available to authorized users.

## Background

Retinoblastoma is the most common primary intraocular tumor in children [1], affecting 11.8 children aged 0–4 years per million in the United States (1975–2004) [2]. External beam radiation therapy has historically been the main therapeutic option for retinoblastoma [3–6], but it can be associated with secondary complications, including radiation-induced cataracts and retinopathy, and malignant neoplasms such as osteosarcomas [7–9]. Therefore, with advances in chemotherapy has come a shift in the therapeutic strategy for retinoblastoma from external beam radiation to systemic chemotherapy. Currently, chemotherapy (carboplatin, etoposide, and vincristine) with local consolidation treatment (laser photocoagulation, cryotherapy and thermotherapy) is the main therapeutic option for retinoblastoma and has contributed to an improvement in patient survival of up to 95 % or more [10, 11].

As the retinoblastoma survival rate has improved, conservation of the eyeball and restoration of visual function have increasingly become important issues, especially in bilateral retinoblastoma. Based on the International Retinoblastoma Classification system, which classifies tumors from A (least severe) to E (most severe), globe salvage rates were found to be 100, 93 and 90 % for eyes classified as A, B and C, respectively [12]. However, in cases of advanced retinoblastoma (groups D and E), the eyeball salvage rate was still low, despite advancements in chemotherapy [12–14]. Various new therapeutic modalities, such as intravitreal chemotherapy and intraarterial chemotherapy via the ophthalmic artery, have been tried to improve eyeball salvage rates [15, 16]. However, these approaches have not proved sufficient to preserve the eyeball in advanced retinoblastoma.

Angiogenesis is essential for the survival, rapid growth, and local invasion of solid tumors [17, 18]. Members of the vascular endothelial growth factor (VEGF) family are known to play critical roles in tumor angiogenesis [19, 20]. A number of studies have reported that blocking VEGF reduces angiogenesis and tumor mass in various cancers [21–23]. Notably, anti-VEGF agents, such as bevacizumab, have been used to treat metastatic cancer by reducing tumor angiogenesis [24–27].

Angiogenesis in retinoblastoma also plays an important role in tumor growth and invasion, and the extent of retinoblastoma angiogenesis could be a prognostic indicator. Specifically, Marback et al. reported that tumor angiogenesis in retinoblastoma is a prognostic factor for disease dissemination, and Arean et al. reported a positive correlation between the intensity of VEGF staining and mitotic and apoptotic indexes [28, 29]. We previously demonstrated that retinoblastoma cells express VEGF mRNA and secrete VEGF protein, which promotes subsequent proliferation of nearby vascular endothelial cells. Additionally, we showed that this enhanced tumor retinoblastoma angiogenesis was significantly reduced and tumor growth was ultimately decreased following bevacizumab treatment [30], Nevertheless, the potential of anti-VEGF treatment in retinoblastoma remains to be demonstrated.

Recently, new reagents targeting VEGF have been developed and approved for the treatment of various tumors and other ocular diseases [31]. Aflibercept (Regeneron, NY, USA), a fusion protein combining the Fc portion of human IgG1 with the principal extracellular ligand-binding domains of human VEGF receptor 1 (VEGFR1) and VEGFR2, acts as a high-affinity, soluble decoy VEGF receptor and potent angiogenesis inhibitor. Preclinical studies have demonstrated potent antitumor and anti-angiogenic activity of aflibercept against a variety of tumors [32, 33].

We hypothesized that aflibercept treatment of retinoblastoma would exert anti-angiogenic and antitumor activities as well as decrease invasive growth to the choroid. Therefore, we sought to evaluate the effects of aflibercept on tumor growth, invasion, and angiogenesis in retinoblastoma using in vivo and in vitro models.

## Methods

### Chemical and reagents

Aflibercept (Regeneron, NY, USA) was provided by Bayer (Leverkusen, Germany). Matrigel Matrix Basement Membrane HC was purchased from BD Biosciences (San Jose, CA, USA).

### Animals

The animal experimental protocol was approved by the Internal Review Board for Animal Experiments of Asan Life Science Institute, University of Ulsan College of Medicine (Seoul, Korea). Female, 4-week-old (14–15 g) athymic nude mice were purchased from Orient Bio Inc. (Seoul, Korea) and maintained at 24 °C ± 0.5 °C under a 12-h light/dark cycle with free access to food and water before and after experiments.

### Y-79 and WERI-Rb-1 human retinoblastoma cell culture

The Y-79 and WERI-Rb-1 human retinoblastoma cell lines were purchased from American Type Culture Collection (ATCC, Manassas, VA, USA). The Y-79 cell line was cultured in Roswell Park Memorial Institute medium (RPMI; Invitrogen, Carlsbad, CA, USA) containing 20 % fetal bovine serum (Invitrogen) and 1 % penicillin-streptomycin (Lonza, Allendale, NJ, USA). The WERI-Rb-1 cell line was cultured in RPMI containing 10 % fetal bovine serum and 1 % penicillin-streptomycin at 37 °C in a humidified 5 % CO_2_ incubator.

### Xenotransplantation model

Four-week-old female athymic nude mice were used for the xenotransplantation model. The animals were injected subcutaneously in the right subaxillary region with 1.5 × 10^7^ Y-79 cells in 0.3 ml of a 1:1 mixture of Matrigel Matrix HC and RPMI with 20 % bovine serum (Invitrogen). Matrigel Matrix HC was used to enhance the success rate of xenografts [34, 35]. One week after subcutaneous Y-79 cell injection, animals were evaluated for successfully transplantation of tumors. Mice in which injected Y-79 cells were successfully transplanted (tumor volume > 200 mm^3^) were divided into two groups and injected intraperitoneally with aflibercept (25 mg/kg) or an equal volume of saline twice weekly for 3 weeks [33, 36–41]. The operator who injected aflibercept or saline was blinded to group assignments. Tumor sizes and body weights of mice were measured once weekly. The longest and shortest dimensions of the tumor were measured, and the volume of each tumor was calculated using the modified ellipsoidal formula [42, 43], where tumor volume = (the longest diameter × the shortest diameter^2^)/2. Four weeks after xenotransplantation, tumors were harvested for tumor weight measurements and immunohistochemical assessments.

### Orthotopic xenotransplantation model

Four-week-old female athymic nude mice were also used for the orthotopic xenotransplantation model. After obtaining a fundus image of each mouse with MICRON III (Phoenix, CA, USA), 1 × 10^5^ (1 μl) Y-79 or WERI-Rb-1 cells were injected into the vitreous using a 30-gauge Hamilton syringe [44–46]. A fundus image of each mouse was also obtained 2 weeks after intravitreal Y-79 or WERI-Rb-1 cells injection to confirm that injected Y-79 or WERI-Rb-1 cells were successfully transplanted. Mice in which injected Y-79 or WERI-Rb-1 cells were successfully transplanted were randomly assigned to two groups: an aflibercept treatment group and a saline treatment group. For the orthotopic transplantation model, intraperitoneal injections of aflibercept or saline were repeated twice weekly for 3 weeks. Five weeks after orthotopic transplantation, a fundus image of each mouse was obtained with MICRON III and the eyeball was enucleated to evaluate choroidal invasion and apoptosis of the retinoblastoma.

### Immunohistochemistry for microvessels

After harvesting, tumor tissue was frozen rapidly and stored at −80 °C. For immunohistochemical examination, tissues were sectioned at 10 μm intervals using a cryostat. After fixation with 4 % paraformaldehyde (PFA), the sections were incubated in permeabilizing and blocking solution composed of phosphate-buffered saline (PBS) containing 1 % bovine serum albumin (BSA) and 0.2 % Triton X-100. Each section was immunostained with isolectin (B4-594; Molecular Probes, Carlsbad, CA, USA), which binds to perivascular cells and endothelial cells.

### Microvascular density analysis in xenotransplantation model

Microvascular density was quantified by analyzing the fraction of lectin-positive pixels per total field using a computer-assisted method [47]. Briefly, the images were binarized to black and white with a common threshold level, such that white pixels represent lectin-positive cells. The fraction of white pixels, representing lectin-stained blood vessels, was automatically quantified by histogram analysis using Adobe Photoshop CS4 (Adobe Inc., Mountain View, CA, USA). For Microvascular density quantification, three fields of each section were used (Additional file 1: Figure S1).

### TUNEL staining

The degree of apoptosis was determined using a terminal dUTP nick-end labeling (TUNEL) kit, as described by the manufacturer (Roche, Basel, Switzerland). Briefly, frozen sections were fixed with 4 % PFA for 20 min at room temperature. After incubated in 0.2 % Triton X-100 in 0.1 % sodium citrate (SSC) buffer, the sections were stained with TUNEL reagent. Fixed cells were then incubated in a nucleotide mixture containing fluorescein-12-dUTP and TdT (terminal transferase) at 37 °C humid chamber for 60 min. Apoptosis of xenotransplantation model was quantified by counting the number of TUNEL-positive cells in four fields of each section (area, 439.377 × 330.769 μm^2^/image) [48, 49]. For quantification of apoptosis in orthotopic xenotransplantation model, three images (two center and one peripheral retina; area, 877.148 × 660.329 μm^2^/image) from largest eyeball section were captured. Number of TUNEL-positive cells were counted and the operator who counted TUNEL-positive cells was blinded to group assignments.

### Quantification of subretinal invasion in the orthotopic xenotransplantation model

The largest eyeball section was used for image analysis in the orthotopic xenotransplantation model. Sections were stained using a standard hematoxylin and eosin (H&E) staining protocol, and the whole eyeball area and the subretinal invasion area were manually traced using ImageJ software. Areas were quantified using the program’s “measure” function, yielding a numerical result in pixels. The amount of subretinal invasion was quantified by calculating the ratio of the subretinal invasion area to the entire eyeball area. The percentage of retinal layer integrity preserved was also analyzed (Additional file 2: Figure S2).

### Retinoblastoma cell and HUVEC co-culture model

To analyze the effect of aflibercept on endothelial cell proliferation in vitro, we conducted studies using human umbilical vein endothelial cells (HUVECs) co-cultured with Y-79 cells or WERI-Rb-1 cells. HUVECs and culture media were purchased from Lonza. The co-culture system was prepared by first plating HUVECs onto 6-well tissue culture plates (0.5 × 10^5^ cells/well) and incubating them for 24 h, at which point HUVECs adhered strongly to the floors of the wells. HUVECs were cultured in Endothelial Growth Media (EGM) on fibronectin-coated plates at 37 °C in a humidified 5 % CO_2_ incubator. After 1 day, the medium was then replaced with a mixed medium of RPMI and EGM (1:1), and Y-79 cells (1 × 10^5^ cells/well) or WERI-Rb-1 cells (1 × 10^5^ cells/well) were added.

### Cell proliferation

The co-cultured Y-79 cells, WERI-Rb-1 cells and HUVECs were treated with 1 mg/ml aflibercept for 3 days. Untreated, co-cultured cells incubated for 3 days served as controls. Three days after aflibercept treatment, proliferation of HUVECs in co-culture with Y-79 or WERI-Rb-1 cells were assessed. After removing Y-79 or WERI-Rb-1 cells, HUVECs were fixed with 4 % PFA for 15 min at room temperature and stained with DAPI (4′,6-diamidino-2-phenylindole). HUVEC proliferation was quantified by counting the number of DAPI-positive cells in four fields of HUVECs (area, 439.377 × 330.769 μm^2^/image).

### Statistical analysis

All results are presented as means ± SEM (standard error of the mean). Student’s t-tests were used to evaluate the significance of differences between two groups. *P*-values < 0.05 were considered statistically significant. SPSS version 21.0 (SPSS, Inc., Chicago, IL, USA) was used to perform all analyses, and graphical presentations were created using Sigma Plot version 10.0 software (Systat Software, Inc., San Jose, CA, USA).

## Results

### Xenotransplantation model

**Fig. 1:**
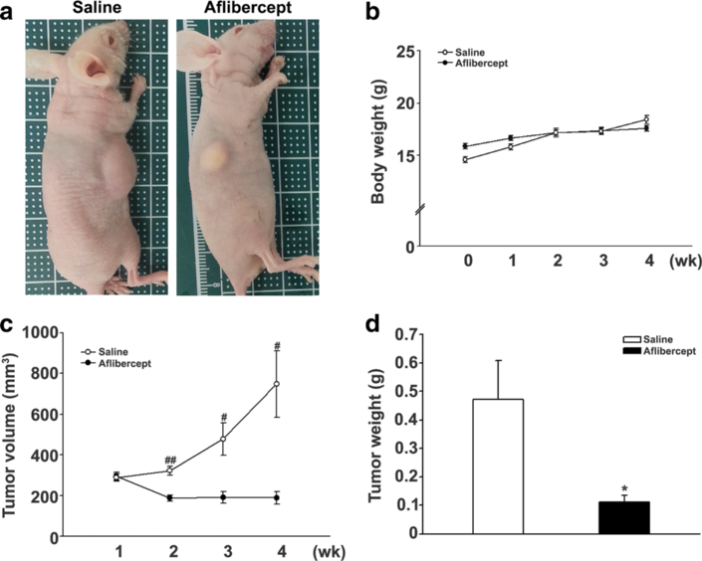
Tumor volume and weight changes in the xenotransplantation model. **a** Representative macroscopic appearance of the xenotransplantation model 4 weeks after injection of Y-79 cells. Tumors were smaller in aflibercept-treated mice (*n* = 21) compared with those in saline-injected group (*n* = 21). **b** Body weight changes in the xenotransplantation model. Four weeks after Y-79 cell injection, body weights were not different between the two groups (**c**) Tumor volume changes in the xenotransplantation model. Whereas tumor volume significantly increased in the saline-injected group (^#^ *P* < 0.005, ^##^ *P* < 0.001), it remained unchanged in the aflibercept (25 mg/kg) treatment group. **d** Tumor weight changes in the xenotransplantation model. Tumor weights in the saline-injected group were also significantly heavier compared to those in the aflibercept treatment group (^*^ *P* < 0.05)

Xenotransplantation model mice, prepared by injecting Y-79 human retinoblastoma cells as described in Materials and Methods, were treated with aflibercept or saline and their tumors were assessed. Macroscopically, retinoblastomas from aflibercept-treated mice appeared smaller than those from saline-injected mice (Fig. 1a). Quantification of tumor volumes confirmed this finding, showing that aflibercept treatment prevented tumor volumes of retinoblastoma from significantly increasing (Fig. 1c). Specifically, at the time mice were divided into subgroups (1 week after Y-79 cell injection), tumor volumes were not different between aflibercept-treated mice (289 ± 71 mm^3^) and saline-injected mice (294 ± 78 mm^3^; *P* = 0.842). After treatment for 3 weeks, tumor volumes in the aflibercept treatment group (189 ± 119 mm^3^) remained unchanged compared with their starting volume (289 ± 71 mm^3^), whereas tumor volumes of saline-injected mice were significantly increased from 294 ± 78 mm^3^ to 748 ± 119 mm^3^. As shown in Fig. 1d, tumor weights in the aflibercept-treated group (0.11 ± 0.08 g) were also significantly less than those in saline-injected mice (0.47 ± 0.51 g; *P* < 0.05). The mean weight change of mice in aflibercept-treated and saline-injected was not different (Fig. 1b).

### Reduced angiogenesis in tumor tissue of mice after aflibercept treatment

**Fig. 2:**
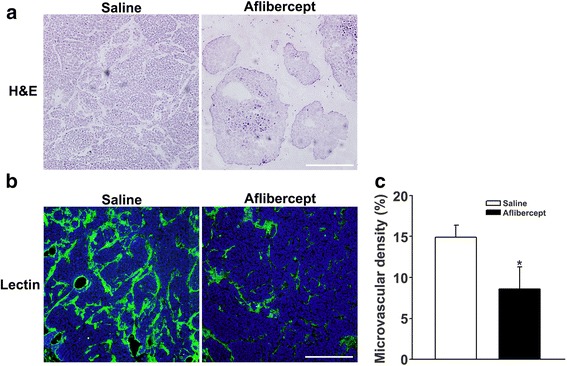
Aflibercept treatment suppresses tumor angiogenesis in a xenotransplantation model. **a** H&E-stained tumor tissue sections. Retinoblastoma cell density was reduced in the aflibercept treatment group compared with that in the saline-injected group. Substantially fewer vascular endothelial cells were detected in the aflibercept-treated group. **b** Representative lectin staining (green) of microvessels in tumor sections. Microvascular density in the aflibercept group was significantly lower than that in the saline-injected group. **c** Quantification of microvascular density (*P < 0.05). Original magnification, ×200; scale bar, 200 μm

H&E staining of tumor tissue obtained 4 weeks after Y-79 cell xenotransplantation (3 weeks after aflibercept or saline treatment) showed that the cell density of xenotransplanted retinoblastomas in the aflibercept-treated group was lower than that in the saline-injected group (Fig. 2a, Additional file 1: Figure S1). There were also substantially fewer vascular endothelial cells within tumor vessels in the aflibercept-treated group (Fig. 2a). To confirm that aflibercept treatment reduced tumor-induced angiogenesis, we stained for blood vessels using lectin. Lectin staining showed that microvascular density in the aflibercept group was less than that in the saline-injected group (Fig. 2b). This was confirmed by a quantitative analysis of lectin-positive cells (Fig. 2c), which showed that the percentage of microvascular coverage in the aflibercept-treated group (8.59 % ± 7.60 %) was significantly less than that (*P* < 0.05) in the saline-injected group (14.91 % ± 4.53 %).

### Increased apoptosis in aflibercept-treated mice

**Fig. 3:**
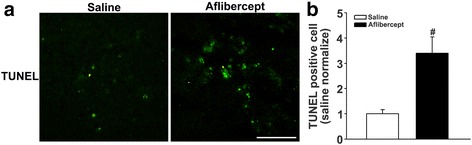
Apoptosis in a xenotransplantation model is increased after aflibercept treatment. **a** Fluorescence photomicrographs of TUNEL staining (green) in tumor tissue sections. **b** Quantification of TUNEL staining. The number of TUNEL-positive cells was significantly increased in the aflibercept group compared with the saline-injected group (^#^ *P* < 0.005). Original magnification, ×400; scale bar, 200 μm

To determine whether the reduction in angiogenesis in tumors after aflibercept treatment ultimately led to an increase in apoptotic cell death of retinoblastoma cells, we performed TUNEL staining. The number of TUNEL-positive cells was increased in the aflibercept treatment group compared with the saline-injected group (Fig. 3a). As shown in Fig. 3b, the number of apoptotic (TUNEL-positive) cells was increased ~2-fold in tumors of aflibercept-treated mice (15.10 ± 9.13) compared with that in saline-injected group (4.44 ± 2.24; *P* < 0.05).

### Reduced subretinal invasion and tumor angiogenesis in the orthotopic xenotransplantation model after aflibercept treatment

**Fig. 4:**
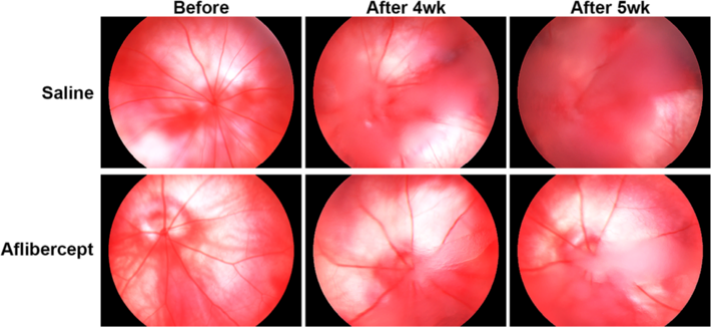
Suppressed Y-79 cell proliferation in the vitreous cavity after aflibercept treatment. As the tumor proliferated in the vitreous cavity, vitreous haziness became worse. 5 weeks after intravitreal injection of Y-79 cells, vitreous haziness which represented Y-79 cells proliferation was worse in the saline-injected group (*n* = 8), compared with that in the aflibercept treatment group (*n* = 14)

**Fig. 5:**
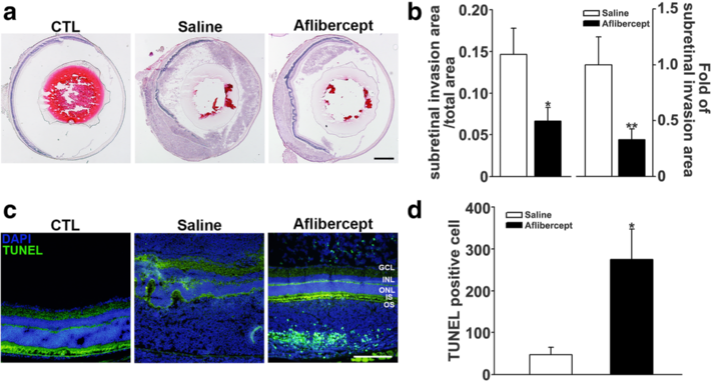
Aflibercept treatment suppressed retinoblastoma subretinal invasion and increased apoptosis in an orthotopic xenotransplantation model. **a** Representative H&E staining of the orthotopic xenotransplantation model. Subretinal invasion sizes were smaller in aflibercept-treated mice (*n* = 14) than in saline-injected group (*n* = 8). Original magnification, ×40; scale bar, 500 μm. **b** Quantification of subretinal invasion. Retinoblastoma subretinal invasion was significantly reduced after aflibercept treatment compared with the saline-injected group (^*^ *P* < 0.05, ^**^ *P* < 0.01). **c** Representative TUNEL staining (green) of the orthotopic xenotransplantation model. TUNEL positive cells were significantly increased in aflibercept treatment group. Original magnification, ×200; scale bar, 200 μm. **d** Quantification of TUNEL positive apoptosis cell number (^*^ *P* < 0.05)

After intravitreal injection, Y-79 tumor cells proliferated in the vitreous cavity, leading to worsening of vitreous haziness (Fig. 4). To determine whether aflibercept treatment reduced subretinal invasion of retinoblastoma, we performed H&E staining and quantification of subretinal invasion (Fig. 5a, Additional file 2: Figure S2). As shown in Fig. 5b, mice in the aflibercept treatment group showed a significant reduction in the subretinal invasion of retinoblastoma (aflibercept-treated, 0.07 ± 0.06; saline-injected, 0.15 ± 0.10, *P* < 0.05). In association with this reduction in subretinal invasion of retinoblastoma, apoptosis was significantly increased (Fig. 5c,d) and retinal layer integrity was preserved in the aflibercept-treated group (12/14 [85.7 %]) compared with the saline-injected group (1/8 [12.5 %] *P* < 0.05). Like Y-79 cells, WERI-Rb-1 cells were well proliferated after intravitreal injection (Additional file 3: Figure S3A). However, unlike orthotopic xenotransplantation model with Y-79 cell, WERI-Rb-1 cell was not invaded into the subretinal space (Additional file 3: Figure S3B). Because the lack of invasiveness of WERI-Rb-1 cells rendered the orthotopic xenotransplantation model unsuccessful, we were unable to use it in the present study. And several previous studies also reported the poor invasiveness of WERI-Rb-1 cells into the subretinal space [50, 51].

### Reduced HUVEC proliferation following aflibercept treatment

**Fig. 6:**
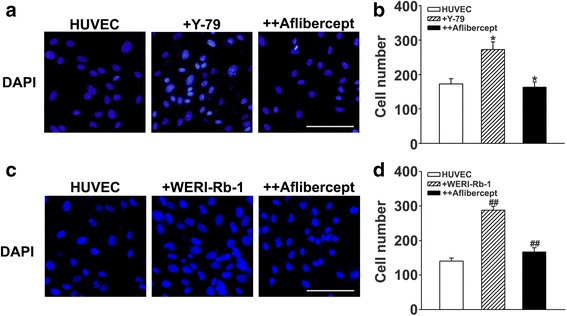
Effect of aflibercept on proliferation of HUVECs co-cultured with Y-79 or WERI-Rb-1 cells. **a** Fluorescence photomicrographs of DAPI staining (blue) in HUVEC co-cultures. Image showing HUVECs alone or HUVECs co-cultured with Y-79, with or without treatment with 1 mg/ml aflibercept. **b** Quantification of cell number. The number of HUVECs was increased by co-culture with Y-79 cells and attenuated by treatment with aflibercept (*n* = 4, ^*^ *P* < 0.05). **c** Fluorescence photomicrographs of DAPI staining (blue) in HUVEC co-cultures. Image showing HUVECs alone or HUVECs co-cultured with WERI-Rb-1, with or without treatment with 1 mg/ml aflibercept. **d** Quantification of cell number. The number of HUVECs was increased by co-culture with WERI-Rb-1 cells and attenuated by treatment with aflibercept (*n* = 4, ^##^ *P* < 0.001, ^*^ *P* < 0.05). Original magnification, ×400; scale bar, 100 μm

To test the effect of aflibercept on retinoblastoma cell-induced vascular endothelial cell proliferation, we conducted an in vitro study used co-cultured HUVECs and Y-79 cells. DAPI staining showed that HUVEC proliferation was increased by co-culture with Y-79 cells, an effect that was attenuated by treatment with aflibercept (Fig. 6a). Quantification of DAPI-stained HUVECs by cell counting confirmed this effect, showing a significant reduction in the proliferation of HUVECs after aflibercept treatment of HUVEC and Y-79 cell co-cultures (Fig. 6b). We also found that WERI-Rb-1 cells increased HUVEC proliferation and WERI-Rb-1 cell induced angiogenesis was also significantly inhibited by aflibercept treatment (Fig. 6c,d).

## Discussion

The primary finding of this study is that aflibercept treatment is capable of inhibiting retinoblastoma tumor growth and invasion. Moreover, aflibercept treatment significantly reduced tumor vessel density and induced apoptosis in the tumor mass. In vitro results further showed that HUVEC proliferation increased when co-cultured with Y-79 cells, an effect that was attenuated by aflibercept treatment. Angiogenesis in retinoblastoma plays an important role in tumor growth and local invasion, and the amount of angiogenesis in the retinoblastoma tumor has been suggested to be a prognostic factor [28, 29]. Therefore, aflibercept treatment could have therapeutic efficacy against retinoblastoma by reducing tumor angiogenesis.

Retinoblastoma is the most common primary intraocular tumor in children [1]. The overall incidence of retinoblastoma in Korea, determined between 1993 and 2010, was 11.2 per 1,000,000 children aged 0 to 4 [52]. If untreated, retinoblastoma can lead to death because of distant metastasis [53, 54]. With advances in systemic chemotherapy, the main therapeutic option for retinoblastoma treatment has become chemotherapy (carboplatin, etoposide, and vincristine) with local consolidation treatment (laser photocoagulation, cryotherapy and thermotherapy), which have contributed to an improvement in patient survival up to 95 % or more [10, 11]. Park et al. reported that the all-cause mortality rate of retinoblastoma in Korea was 7.9 % at 5 years and 8.4 % at 10 years [52].

With this improvement in the survival rate of retinoblastoma patients, globe salvage has become an important issue in retinoblastoma treatment. In advanced-stage retinoblastoma (Group D and E), globe salvage rate has remained low. Okimoto et al. reported that, despite systemic chemotherapy, the globe salvage rate of group D was 33.0 %, and both Okimoto et al. and Naseripour et al. [13, 14] reported a globe salvage rate of 0 % for group E retinoblastoma. To improve the rate of globe salvage, researchers have tested new therapeutic modalities, such as intravitreal chemotherapy and intraarterial chemotherapy via ophthalmic artery [15, 16]. However, those methods were not enough to save the eyeball in advanced retinoblastoma.

Subretinal and optic nerve infiltration of retinoblastoma are cited as the most common causes of failure to save the eyeball after systemic chemotherapy [15, 16]. And angiogenesis might be essential for invasion of retinoblastoma into the subretinal space or optic nerve [28], and several studies have reported a relationship between angiogenesis and tumor invasion or disease dissemination [28, 55–57]. Therefore, if retinoblastoma angiogenesis could be blocked, it should be possible to reduce subretinal or optic nerve infiltration of retinoblastoma, which eventually might improve the globe salvage rate.

Previous studies have demonstrated that retinoblastoma cells express VEGF in hypoxic and normoxic conditions [29, 30, 58]. Moreover, HUVEC proliferation is increased by co-culture with Y-79 cells and reduced by bevacizumab treatment [30]. In vitro experiments performed in the current study also evaluated the anti-angiogenic effects of aflibercept on co-cultured HUVECs and Y-79 retinoblastoma cells, demonstrating that aflibercept also reduced HUVEC proliferation in HUVEC and Y-79 retinoblastoma cell co-cultures. Thus, aflibercept treatment is capable of reducing retinoblastoma-induced angiogenesis in vitro.

In the current study, we found that aflibercept treatment reduced tumor growth and invasion to the choroid. To determine whether the suppressed tumor growth and invasion after aflibercept treatment resulted from decreased tumor angiogenesis, we assessed the extent of angiogenesis in the xenotransplanted retinoblastoma after aflibercept treatment by performing immunohistochemical staining with the vascular endothelial cell marker, lectin. These experiments showed a substantial decrease in lectin-positive cells within tumors after aflibercept treatment compared with the saline-injected group. These findings indicate that injected aflibercept reduced angiogenesis within the retinoblastoma, ultimately decreasing tumor volume. We also found that subretinal invasion of the orthotopic xenotransplantation model was significantly reduced after aflibercept treatment. Decreased subretinal invasion after aflibercept treatment reflected that aflibercept treatment could actually decrease subretinal invasion in the eyeball, which leaded to improve glove salvage rate.

TUNEL staining, performed to evaluate the effect of the aflibercept-induced decrease in angiogenesis on the extent of apoptosis in the tumor mass, showed that this decrease in angiogenesis was associated with an increase in apoptosis. Similar results were obtained by Theodoropoulou et al., who reported that aminoimidazole carboxamide ribonucleotide (AICAR) treatment decreased tumor angiogenesis in association with an increase in tumor cell apoptosis [59]. Although it is reasonable to suppose that reduced angiogenesis in the xenotransplanted tumor would increase apoptosis and eventually prevent tumor enlargement, how aflibercept treatment of retinoblastoma increases apoptosis is currently unknown. Notable in this context, aflibercept was not cytotoxic toward retinoblastoma cells. Instead, aflibercept reduced the growth and invasion of retinoblastoma by reducing angiogenesis. Therefore, we assume that, by reducing tumor angiogenesis in the retinoblastoma, aflibercept treatment could have an adjuvant role together with systemic chemotherapy. Accordingly, we would expect combined treatment with systemic chemotherapy and aflibercept to decrease optic nerve and choroidal invasion by reducing angiogenesis, ultimately improving the rate of eyeball salvage. However, considering the fact that such malignancies as colon, or breast cancer, that responded well to initial anti-VEGF treatment, eventually developed anti-VEFG resistance, a long-term anti-VEGF treatment to retinoblastoma could potentially show anti-VEGF resistance. Therefore, long-term anti-angiogenic effect of aflibercept to retinoblastoma should be further evaluated [60].

## Conclusion

We found that aflibercept reduced retinoblastoma angiogenesis in vitro as well as in in vivo xenotransplantation models. As a result of the reduction in retinoblastoma angiogenesis after aflibercept injection, tumor volume did not significantly increase in the xenotransplantation model and tumor invasion decreased in the orthotopic xenotransplantation model. Therefore, aflibercept treatment in retinoblastoma could play an adjuvant role in reducing tumor size and invasion when combined with systemic chemotherapy.

## Additional files

Additional file 1: Figure S1. Automated quantification of angiogenesis in the xenotransplantation model. Upper row showed Lectin staining (green) of microvessels in tumor sections. Lower row represented binarized image (black and white) of Lectin staining. Images were binarized to black and white with a common threshold level, such that white pixels represent lectin-positive cells. The fraction of white pixels, representing lectin-stained blood vessels, was automatically quantified by histogram analysis using Adobe Photoshop CS4. (TIF 706 kb)
Additional file 2: Figure S2. Quantification of subretinal invasion in the orthotopic xenotransplantation model. Eyeball sections were stained using a standard hematoxylin and eosin (H&E) staining protocol, and the whole eyeball area and the subretinal invasion area were manually traced using ImageJ software. Areas were quantified using the program’s “measure” function, yielding a numerical result in pixels. The amount of subretinal invasion was quantified by calculating the ratio of the subretinal invasion area to the entire eyeball area. (TIF 1041 kb)
Additional file 3: Figure S3. In Vivo and In vitro experiment with WERI-Rb-1 cell. (A) MICRON image of orthotopic xenotransplantation model with WERI-Rb-1 cells. Five weeks after WERI-Rb-1 cell intravitreal injection, vitreous haziness became worse. (B) Representative H&E staining of the orthotopic xenotransplantation model with WERI-Rb-1 cell. Though WERI-Rb-1 cell was proliferated in the vitreous cavity, there was no subretinal invasion in the orthotopic xenotransplantation model with WERI-Rb-1 cell (saline = 9, aflibercept = 13). (TIF 2898 kb)

## Abbreviations

H&E: Hematoxylin and eosin
HUVECs: Human umbilical vein endothelial cells
PBS: Phosphate-buffered saline
PFA: Paraformaldehyde
TUNEL: Terminal dUTP nick-end labeling
VEGF: Vascular endothelial growth factor
