# Supplementary figures and images for: Efficacy and safety of aflibercept in in vitro and in vivo models of retinoblastoma
Source: J Exp Clin Cancer Res. 2016 Nov 4;35:171. doi: 10.1186/s13046-016-0451-7 (PMC5097437; doi:10.1186/s13046-016-0451-7)

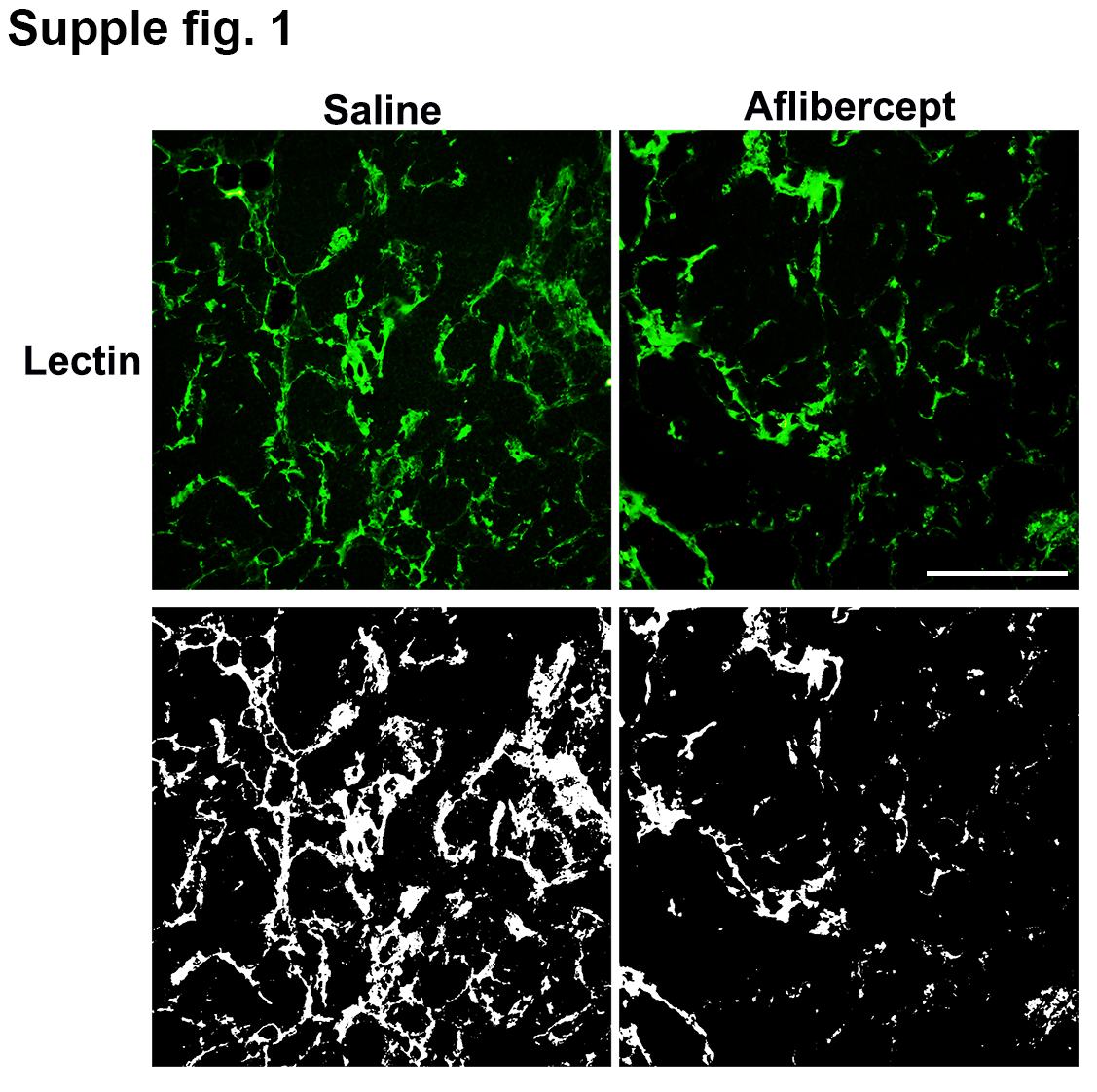

Supplement: Additional file 1: Figure S1. — Automated quantification of angiogenesis in the xenotransplantation model. Upper row showed Lectin staining (green) of microvessels in tumor sections. Lower row represented binarized image (black and white) of Lectin staining. Images were binarized to black and white with a common threshold level, such that white pixels represent lectin-positive cells. The fraction of white pixels, representing lectin-stained blood vessels, was automatically quantified by histogram analysis using Adobe Photoshop CS4. (TIF 706 kb) [file 13046_2016_451_MOESM1_ESM.tif]

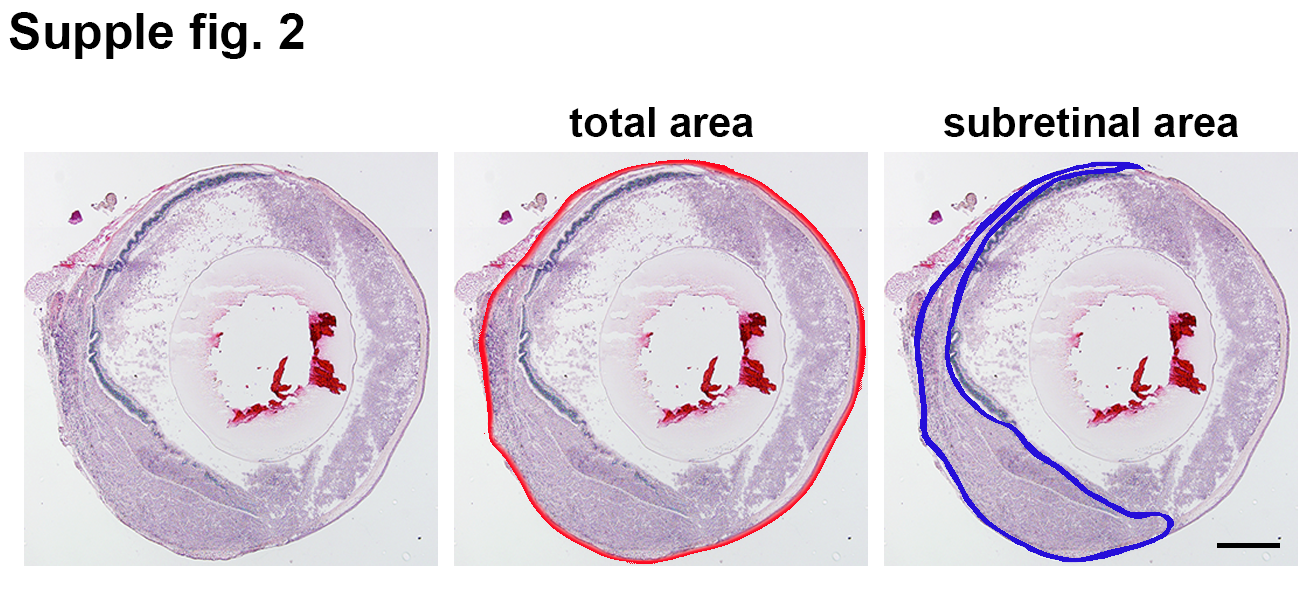

Supplement: Additional file 2: Figure S2. — Quantification of subretinal invasion in the orthotopic xenotransplantation model. Eyeball sections were stained using a standard hematoxylin and eosin (H&E) staining protocol, and the whole eyeball area and the subretinal invasion area were manually traced using ImageJ software. Areas were quantified using the program’s “measure” function, yielding a numerical result in pixels. The amount of subretinal invasion was quantified by calculating the ratio of the subretinal invasion area to the entire eyeball area. (TIF 1041 kb) [file 13046_2016_451_MOESM2_ESM.tif]

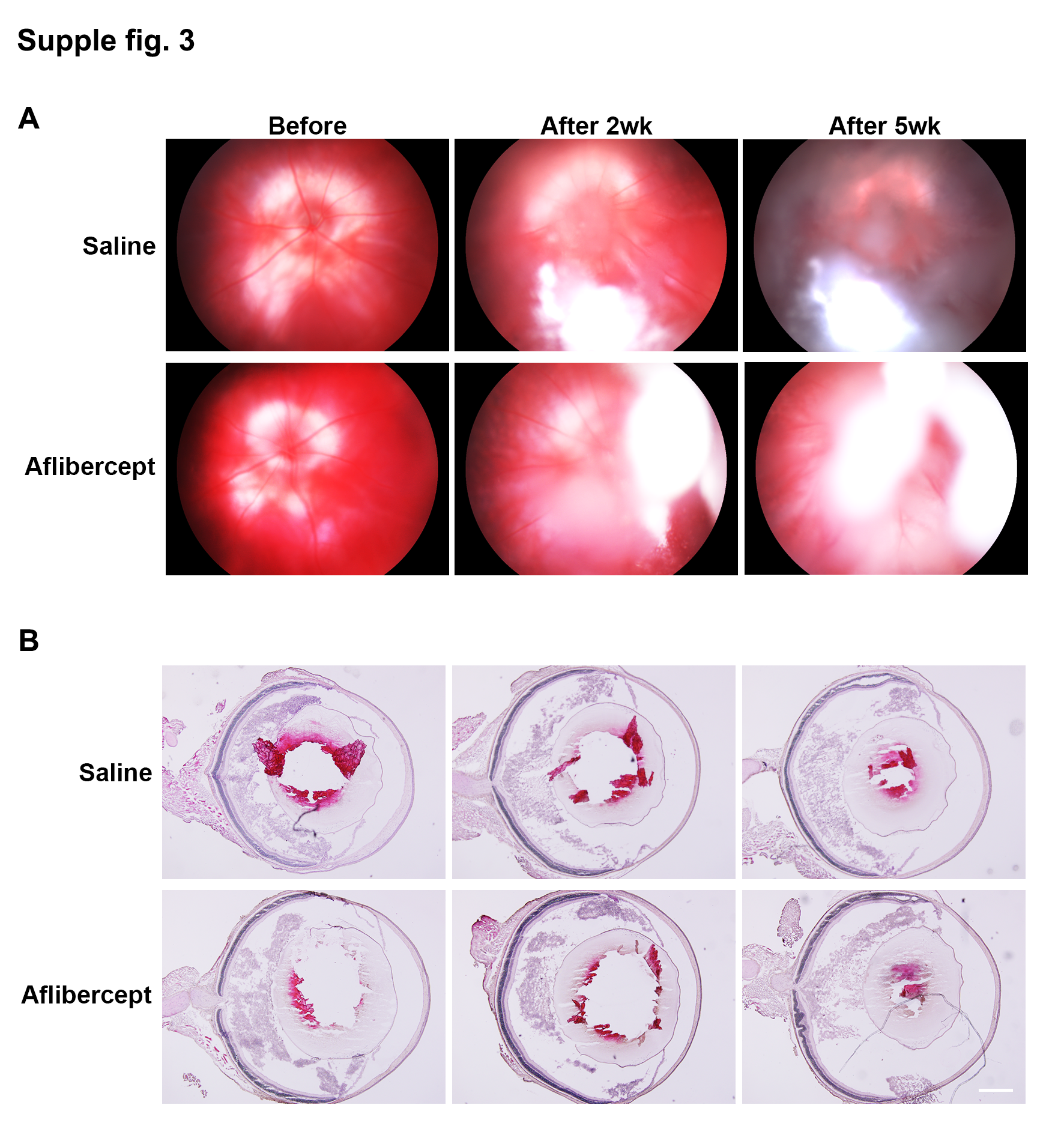

Supplement: Additional file 3: Figure S3. — In Vivo and In vitro experiment with WERI-Rb-1 cell. (A) MICRON image of orthotopic xenotransplantation model with WERI-Rb-1 cells. Five weeks after WERI-Rb-1 cell intravitreal injection, vitreous haziness became worse. (B) Representative H&E staining of the orthotopic xenotransplantation model with WERI-Rb-1 cell. Though WERI-Rb-1 cell was proliferated in the vitreous cavity, there was no subretinal invasion in the orthotopic xenotransplantation model with WERI-Rb-1 cell (saline = 9, aflibercept = 13). (TIF 2898 kb) [file 13046_2016_451_MOESM3_ESM.tif]
